# Adult Intussusception Secondary to Varicella-Zoster Virus Infection

**DOI:** 10.7759/cureus.82204

**Published:** 2025-04-13

**Authors:** Jennifer Madukaife, Micah Pippin, George Collins, Diahann Marshall

**Affiliations:** 1 Family Medicine, Louisiana State University Health Sciences Center, Alexandria, USA; 2 Family Medicine, Rapides Regional Medical Center, Alexandria, USA; 3 Pathology, Rapides Regional Medical Center, Alexandria, USA

**Keywords:** adult small bowel intussusception, atypical varicella, varicella vaccine, varicella-zoster (chickenpox), varicella zoster virus infection

## Abstract

Varicella-zoster virus (VZV), also known as human herpesvirus 3, is a double-stranded linear DNA virus. It can manifest as chickenpox in childhood and, much less commonly, in adulthood, and the latent virus can reemerge as shingles. This case investigates an unusual presentation of intussusception, telescoping of the bowel, in the context of varicella infection. Reports on this phenomenon are very rare, and the effects of VZV on gastrointestinal pathology are primarily theoretical. This case underscores the importance of early diagnosis and targeted management and the need for further investigation into the association between VZV and intussusception.

## Introduction

Varicella-zoster virus (VZV) is responsible for diverse clinical manifestations, ranging from mild self-limited rashes in children to severe complications in immunocompromised individuals and adults [1-2]. Although rare, intussusception is a gastrointestinal complication potentially associated with varicella and has been reported in the literature [1,3]. Previous works have suggested methods by which viral infections may trigger intussusception and have investigated the presence of VZV in affected bowel tissue [3]. Virally induced lymphadenopathy may serve as a lead point for developing intussusception, and neuropathic intestinal sequelae may cause asynchronous peristalsis [3]. While it is accepted that VZV can cause intussusception, there is limited data on the occurrence, especially in adults, and further investigation is needed. This report is a rare case of acute adult VZV infection and concomitant intussusception and adds to the available scholarship on the phenomenon.

## Case presentation

**Figure 1:**
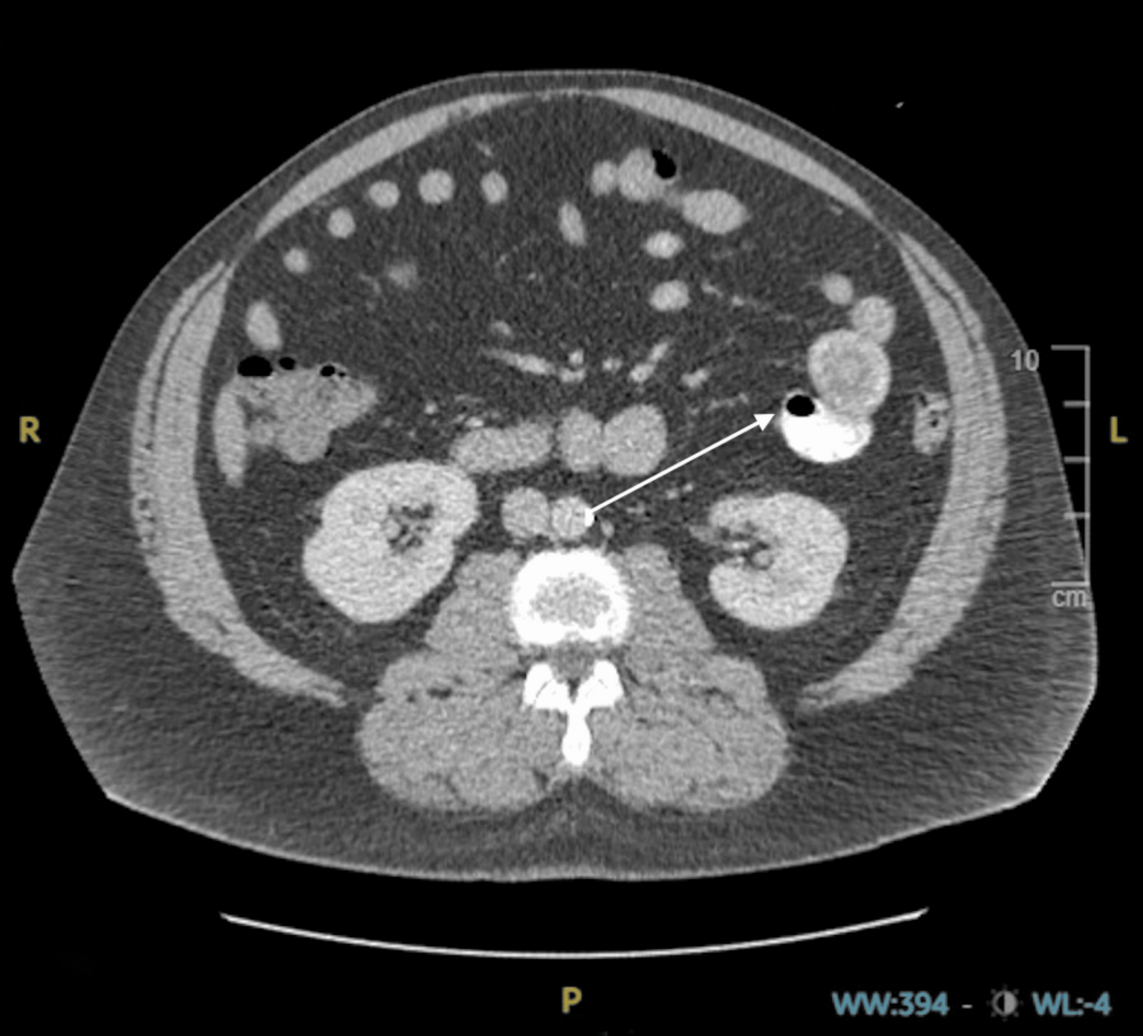
CT scan of the abdomen demonstrating small bowel to small bowel intussusception CT: computed tomography

A 49-year-old male with a past medical history of hypertension, well-controlled diabetes mellitus type 2, hyperlipidemia, and coronary artery disease status post coronary artery bypass grafting two years prior presented to an emergency department complaining of left upper quadrant abdominal discomfort. The patient's symptoms began five days prior to admission, with the foremost symptom being abdominal pain, which radiated to the left flank. The pain was intermittent and ranged from four to nine out of ten on the numeric pain rating scale, with associated nausea and diarrhea. The patient did not report any episodes of emesis. No home analgesics were tried to alleviate the pain, and nothing exacerbated his symptoms. The patient was currently taking enalapril 20 mg orally every day, atorvastatin (Lipitor) 40 mg orally daily, metformin 500 mg orally twice a day, and carvedilol (Coreg) 6.25 mg orally daily. He endorsed a 30-pack-year tobacco smoking history and reported abstinence from alcohol for two years, before which he drank one to two standard servings of alcohol weekly. Evaluation at an outlying facility included an abdominal CT scan without contrast, demonstrating no acute pathology. Initial management included an intravenous lactated Ringer's fluid bolus and antibiotic administration with pharmacy-dosed intravenous piperacillin/tazobactam (Zosyn) and 500 mg of intravenous metronidazole (Flagyl) for presumed acute diverticulitis or pancreatitis. During that admission, the patient developed a widespread rash beginning on the head and spreading caudally toward the chest and upper back. A repeated CT scan with intravenous contrast revealed a possible small bowel intussusception (Figure 1).

**Figure 2:**
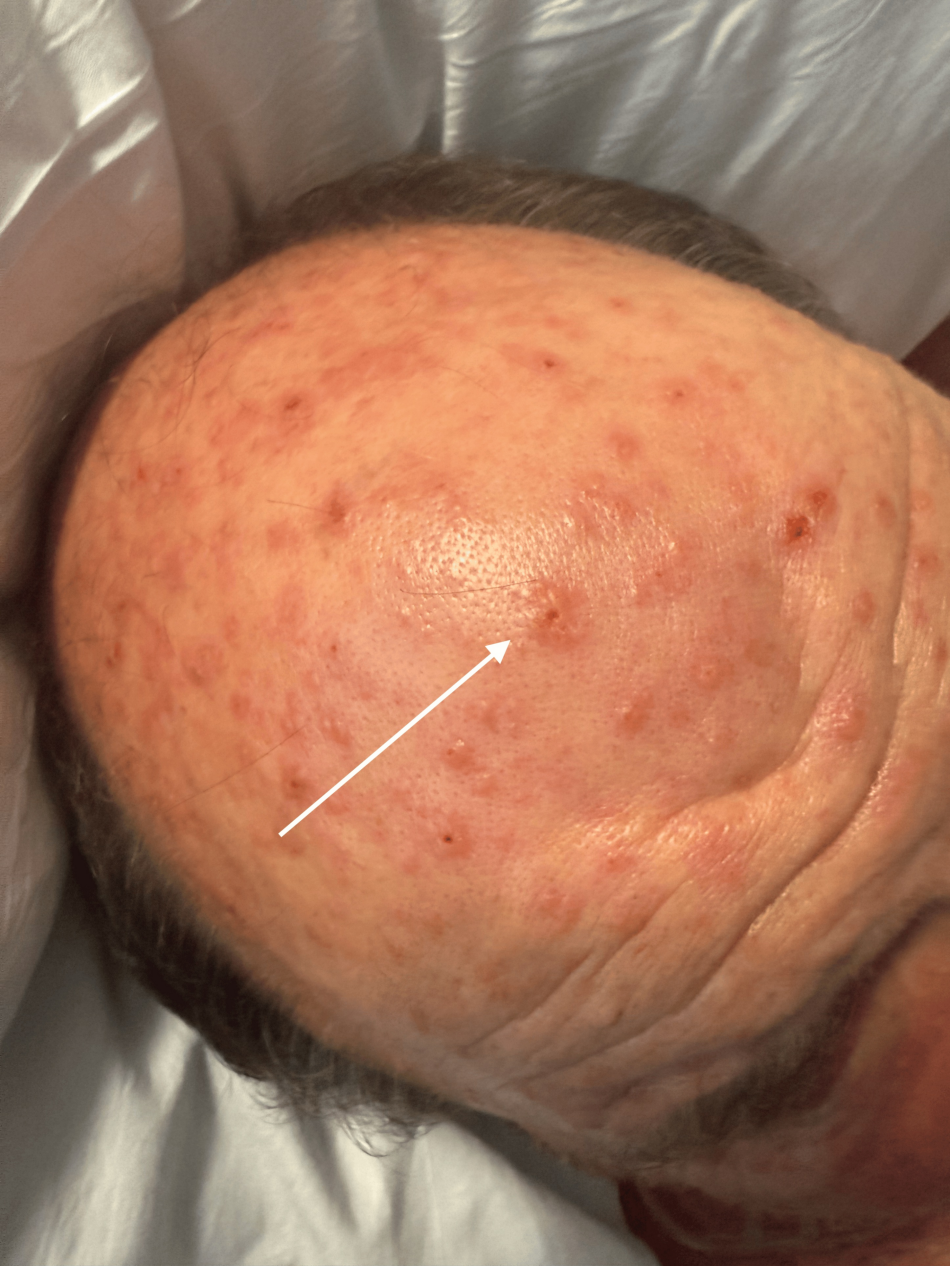
VZV eruption on the patient's scalp VZV: varicella-zoster virus

**Figure 3:**
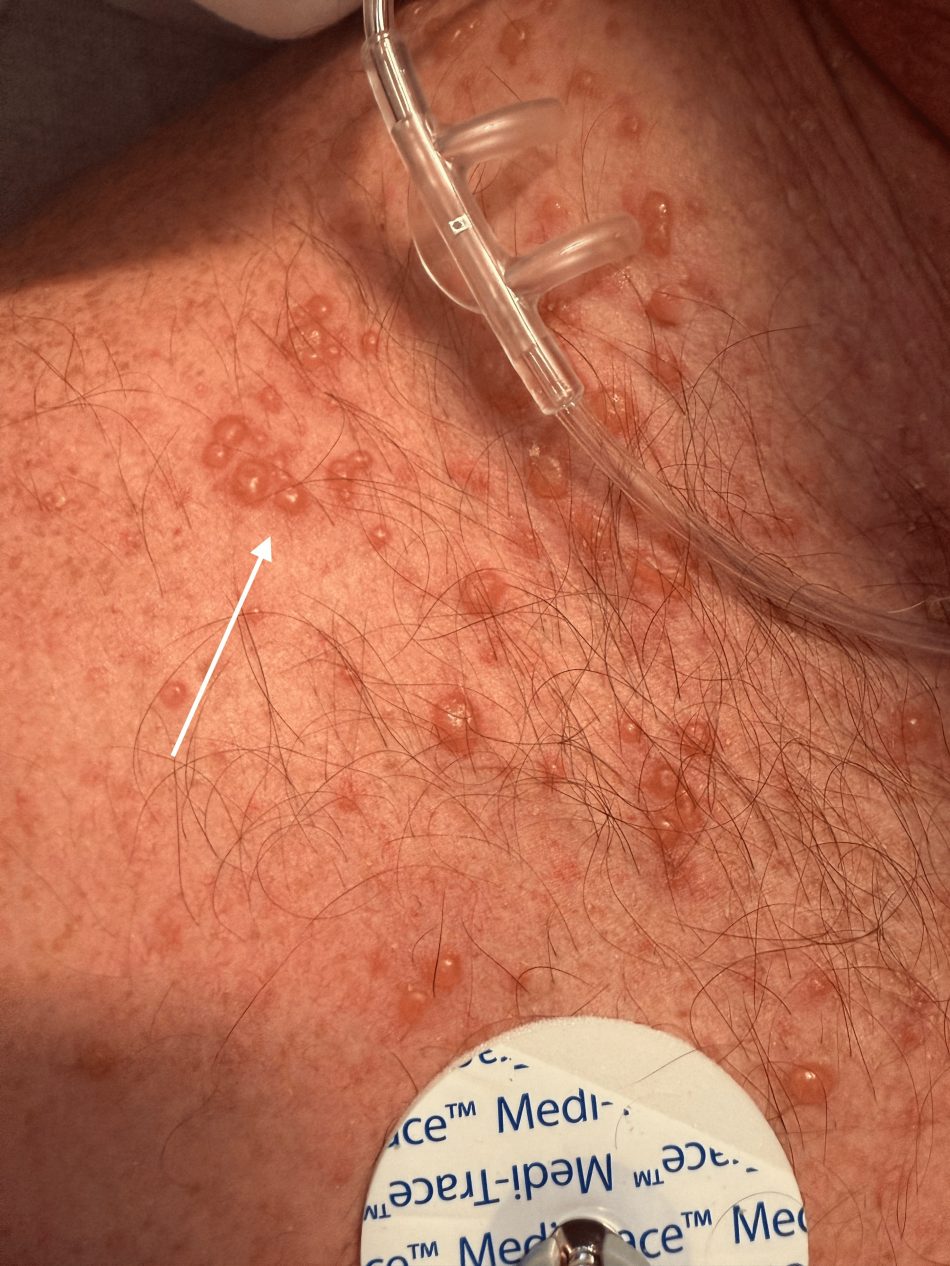
VZV eruption on the patient's chest VZV: varicella-zoster virus

Due to his worsening abdominal pain and new skin eruption, the patient left the hospital against medical advice and presented to our emergency department. Vital signs on presentation included a temperature of 98.2 degrees Fahrenheit, an elevated heart rate of 95 beats per minute, a high blood pressure of 169/77 mmHg, respirations of 18 breaths per minute, and oxygen saturation of 97% on room air by pulse oximetry. The patient weighed 117 kilograms or 258 pounds. Initial physical examination demonstrated a regular heart rate and rhythm, decreased breath sounds bilaterally with no wheezing or rales auscultated, left upper and lower abdominal quadrant tenderness without rebound or guarding, decreased bowel sounds, no peripheral edema, palpable distal pulses to upper and lower extremities bilaterally, and a maculovesicular rash with lesions measuring 0.5-2cm with confluence and distribution to the scalp, neck, upper back, chest, and upper abdomen. The lesions demonstrated clear-draining vesicles on an erythematous base with multiple excoriations (Figures 2-3).

The eruption was not confined to one zone but was diffusely spread, crossing multiple dermatomes. Laboratory evaluation revealed mild leukopenia with elevated monocytes, lymphopenia with atypical lymphocytes, and thrombocytopenia with decreased platelet morphology, all of which can be seen in acute viral infections. A complete metabolic panel resulted in transaminitis and mild hyperbilirubinemia. Serology workup demonstrated negative hepatitis antibodies, negative Epstein-Barr virus antigens, and positive varicella IgG and IgM antibodies (Table 1).

**Table 1:** Complete blood count, complete metabolic panel, hepatitis panel, HIV antibody, Epstein-Barr virus and VZV antibodies HIV: human immunodeficiency virus, VZV: varicella-zoster virus, CO2: carbon dioxide, IgM: immunoglobulin M, IgG: immunoglobulin G

Laboratory analysis	Reported value	Normal range
White blood cells	3.9 x 10⁹/L	5-10 x 10⁹/L
Hemoglobin	15.8 gm/dL	12.0-16.0 gm/dL
Hematocrit	44.9%	40.0-54.0%
Mean corpuscular volume	92.4 FL	80.0-100.0 FL
Mean corpuscular hemoglobin	32.5 PG	26.0-32.0 PG
Red cell distribution width	12.3%	11.5-14.5%
Platelet count	68.0 x 10⁹/L	150-450 x 10⁹/L
Monocytes	16.0%	2-11%
Neutrophils	58%	40-70%
Lymphocytes	17%	20-45%
Eosinophils	0%	0-5%
Basophils	0.0%	0-2%
Bands	9.0%	0-5%
Sodium	134 mmol/L	135-148 mmol/L
Potassium	4.3 mmol/L	3.3-5.1 mmol/L
Chloride	96 mmol/L	98-107 mmol/L
CO2	29 mmol/L	21-32 mmol/L
Anion gap	9	0-15
Glucose	154 mg/dL	70-120 mg/dL
Blood urea nitrogen	11 mg/dL	6-19 mg/dL
Creatinine	0.77 mg/dL	0.70-1.30 mg/dL
Calcium	8.2 mg/dL	8.4-10.7 mg/dL
Aspartate aminotransferase	188 U/L	0-37 U/L
Alanine aminotransferase	137 U/L	0-40 U/L
Alkaline phosphatase	87 U/L	40-130 U/L
Lactic acid	1.6 mmol/L	0.5-2.2 mmol/L
Total bilirubin	1.8 mg/dL	0-1.0 mg/dL
Albumin	4 g/dL	3.2-5.2 g/dL
Lipase	13 U/L	16-63 U/L
Hepatitis A IgM	Negative	Negative
Hepatitis B surface antigen	Negative	Negative
Hepatitis B core IgM	Negative	Negative
Hepatitis C antibody	Negative	Negative
HIV 1 and 2 antibody	Negative	Negative
Epstein-Barr virus capsid antigen IgG	608 U/mL	0-21.9 U/mL
Epstein-Barr virus capsid antigen IgM	<10 U/mL	0-43.9 U/mL
Epstein-Barr virus nuclear antigen IgG	>600 U/mL	0-21.9 U/mL
VZV IgG	1.2 signal/cutoff	<1.0 signal/cutoff
VZV IgM	5.24 ISR	<0.9 ISR

**Figure 4:**
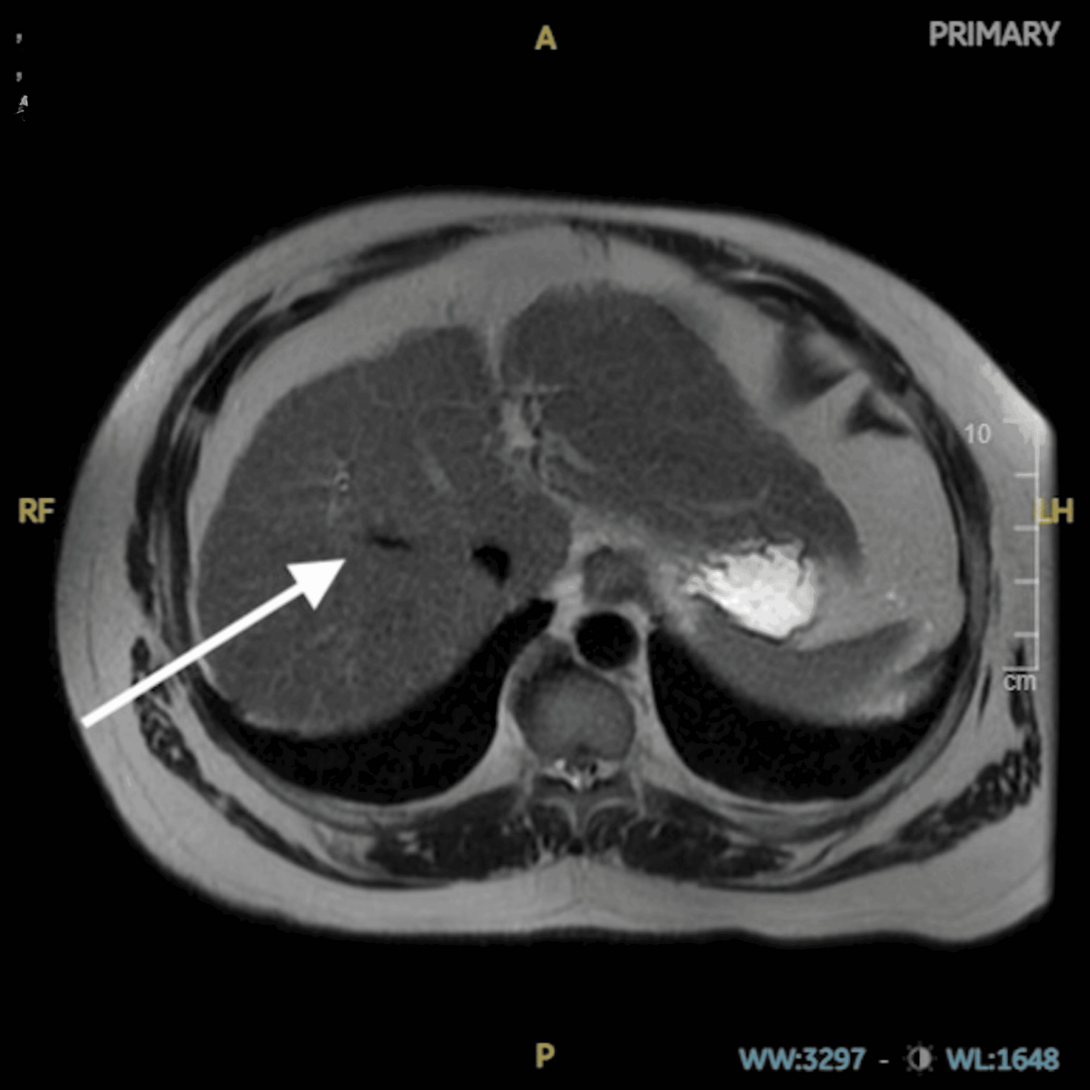
MRI showing liver echogenicity MRI: magnetic resonance imaging

The patient reported never having the varicella or shingles vaccine and did not recall having chickenpox as a child. Due to his transaminitis and elevated bilirubin, a right upper quadrant ultrasound was performed, followed by an abdominal MRI with and without contrast. Both images displayed hepatic echogenicity, suggesting fatty infiltration of the liver, consistent with his history of dyslipidemia and metabolic syndrome (Figure 4).

**Figure 5:**
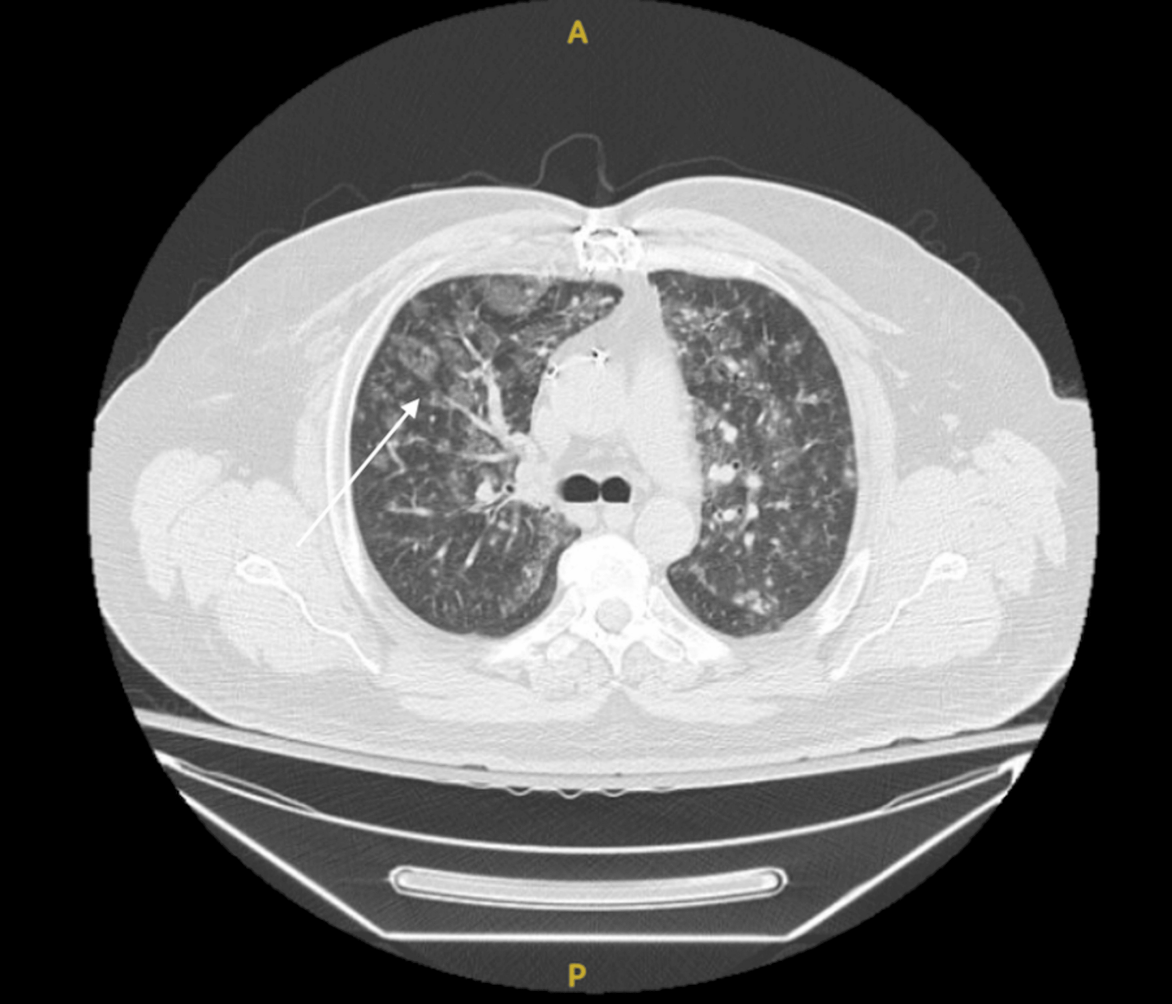
CT scan of the chest demonstrating ground-glass opacities CT: computed tomography

No choledocholithiasis or other biliary obstruction was visualized. A chest CT with intravenous contrast demonstrated reticulonodular and ground-glass opacities, signifying possible pneumonia (Figure 5).

**Figure 6:**
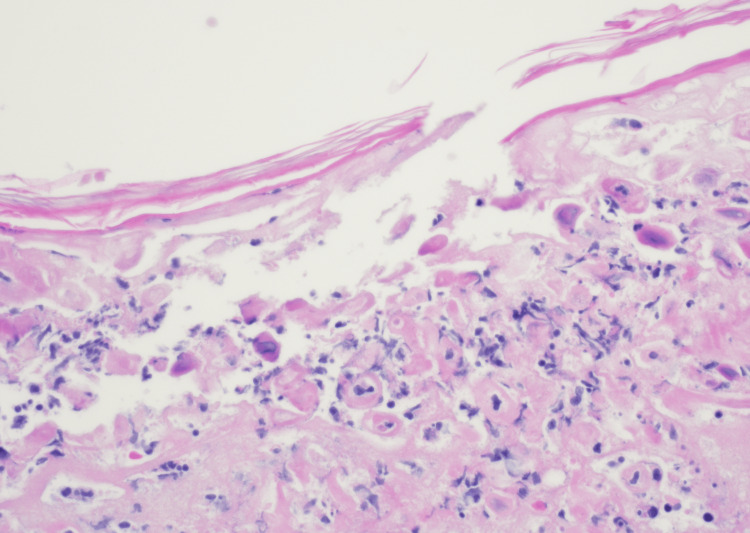
Skin biopsy showing suppurative inflamed intraepidermal vesiculation, acantholysis, ballooning degeneration of keratinocytes, variably multinucleate and uninucleate giant cells, and nuclear chromatin margination

The patient was evaluated by general surgery, who recommended against any operative interventions but for supportive care with intravenous hydration and nothing by mouth as the intussusception resolves. Infectious disease was consulted for further evaluation of the rash, which was concerning for possible varicella, Mpox, or antibiotic-associated hypersensitivity. Two 3 mm skin punch biopsies were collected from lesions on the patient's upper back. Acyclovir 10 mg/kg every eight hours intravenously was initiated due to concern for varicella, along with intravenous vancomycin titrated to target troughs between 10-20 mcg/mL, diphenhydramine 25 mg every eight hours orally, and prednisone 20 mg every day orally. The biopsy resulted in findings suggestive of a herpes viral infection, including suppurative inflamed intraepidermal vesiculation, acantholysis, ballooning degeneration of keratinocytes, variably multinucleate and uninucleate giant cells, and nuclear chromatin margination (Figure 6).

**Figure 7:**
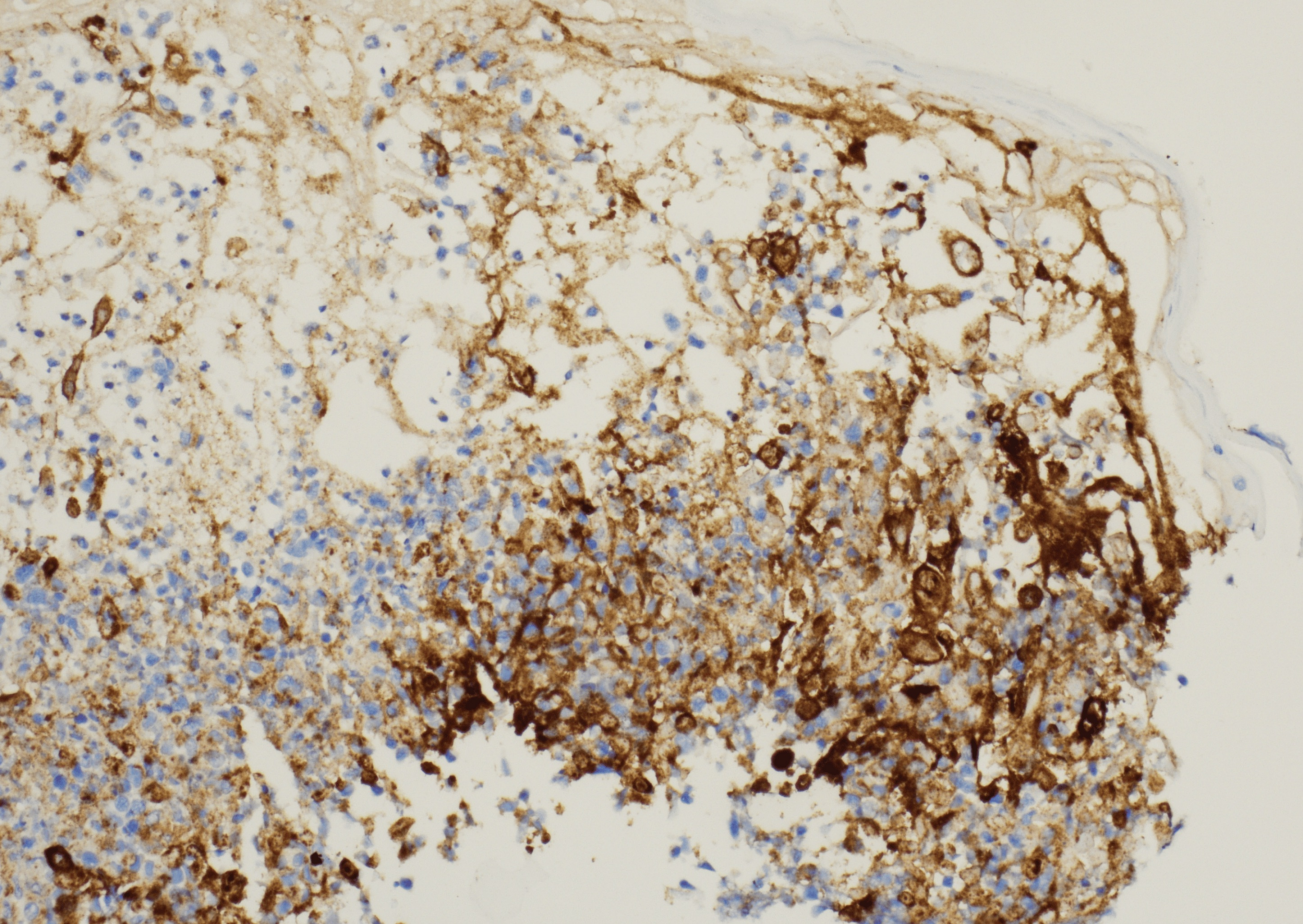
Skin biopsy with positive immunohistochemical staining for VZV VZV: varicella-zoster virus

Immunohistochemical staining was also positive for VZV (Figure 7).

The patient's rash and intussusception improved after administering appropriate antivirals and conservative management. Laboratory abnormalities, including leukopenia, lymphopenia, monocytosis, and thrombocytopenia, all resolved. The patient was subsequently discharged from this facility with a scheduled follow-up with his primary care physician.

## Discussion

This case describes intussusception as a rare complication of varicella infection. VZV, also known as human herpes virus 3, is the causative agent of chickenpox [2,4]. In the United States, in the period spanning 1980-2000, the incidence of varicella infection annually was approximately 15 per 1,000 people [5]. Following approval by the U.S. Food and Drug Administration for routine immunization against varicella, with implementation as of 1996 and additional schedule modifications in 2005, the incidence of cases had decreased to 0.4 per 1000 people by 2005 [4-6]. In the 1990s in the United States, the most significant age-specific incidence of acute varicella infection was among children one to four years of age, accounting for 39% of cases [4]. This predominant age distribution was likely attributed to children of this age attending preschool and childcare centers. There is no described gender or racial predominance for infection rates; however, it has been observed that there is a temporal pattern wherein countries with temperate climates primarily observe children infected by the age of 10 versus tropical areas that will observe infections in children of older ages and some young adults [4]. Varicella has no observed zoonotic transmission and is solely transmitted by humans via contact with vesicular fluids or inhalation of aerosolized respiratory tract secretions [4]. Acute varicella infection typically presents as a prodrome of fever and malaise spanning one to two days prior to the development of a generalized, pruritic rash disseminating typically within 24 hours from the scalp and downwards to the face, trunk, and then extremities in a series of evolving macular to papular and then vesicular lesions, on an erythematous base, before finally crusting [2,4]. Most childhood infections in the healthy resolve within two to four days; however, adults and immunocompromised individuals have been observed to develop a more complicated clinical course, including high fever, extensive vesicular eruption, secondary bacterial infections, pneumonia, encephalitis, hemorrhagic varicella, cerebellar ataxia, Reye syndrome, thrombocytopenia, purpura fulminans, glomerulonephritis, hepatitis, uveitis, myocarditis, arthritis, transverse myelitis, Guillain-Barré syndrome, and intussusception [2,4]. The diagnosis of acute varicella is mostly clinical; however, laboratory evaluation of presumed infection is often sought in severe or unusual clinical courses of the disease in the form of polymerase chain reaction testing of the vesicular secretions or crusted lesions, both serving as adequate specimens [2,4]. Treatment of healthy young patients who contract VZV primarily focuses on symptomatic management, including antipyretics for fever and topical preparations to relieve pruritus [2]. In children, antiviral administration with agents such as acyclovir is minimally effective and only decreases symptoms by one day if taken within 24 hours of rash eruption [2,4]. For this reason, antivirals are generally not recommended for typical childhood infections. Due to the more severe nature and possible complications of acute varicella in adults, antivirals may be recommended in addition to supportive care to improve prognosis [2].

Shingles, otherwise known as herpes zoster, is also caused by human herpes virus 3; however, it is a distinct clinical entity from acute varicella infection [7]. Shingles is a reactivation of latent varicella virus in adults, which persists in the dorsal root ganglia following primary varicella infection [5,7]. Risk factors for shingles include immunosuppression, previous infection with varicella at younger than 18 months of age, and older age [5,7]. While varicella’s dermatologic manifestation is generally diffuse, the shingles rash is often confined to a single dermatome or nerve pathway [7]. It is frequently preceded by a prodrome of pain, numbness, and tingling before erupting in vesicular lesions on erythematous bases, which crust as they progress [5,7]. Severe pain is associated with the shingles outbreak instead of the mainly pruritic chickenpox rash. Treatment of shingles is focused on decreasing the duration and severity of symptoms by administering antivirals and emphasizing proper analgesic management. Postherpetic neuralgia and other neurologic sequelae are common complications of shingles and ocular, pulmonary, and hepatic manifestations [7]. Just as acute varicella infection has been associated with intussusception, so has its shingles counterpart [8].

Immunization has been paramount in preventing varicella infection and its complications in children and adolescents. Varivax, a live attenuated virus vaccine, or MMRV, a combination injection against measles, mumps, rubella, and varicella, is administered at 12 to 15 months of age and again at four to six years of age [4-6,9]. Following exposure to varicella in patients 12 months of age and older who are not previously vaccinated or do not have evidence of immunity, a post-exposure prophylaxis dose of the varicella vaccine is recommended, ideally within three to five days of exposure [5-6]. In individuals at high risk of severe varicella infection and its complications, such as immunocompromised individuals, a VZV immune globulin dose can be administered following exposure [5-6]. Pregnant women may also receive immune globulin following varicella exposure since the live attenuated virus vaccine is contraindicated [5]. To prevent reactivation of the VZV virus in adults, also known as herpes zoster or shingles, vaccination with Shingrix is recommended for all patients 50 years of age and older [5-6]. Shingrix is a recombinant subunit protein vaccine given in two doses, two to six months apart, and has replaced the previous Zostavax, a live attenuated vaccine [4-6]. While Shingrix is typically reserved for older patients, recommendations suggest administration in immunosuppressed patients 19 years of age and above [5-6]. Outside of immunization, the isolation of afflicted individuals prevents transmission to healthy individuals until no new lesions appear within the past 24-hour period [4].

Intussusception is the telescoping of a segment of bowel into an adjacent section. Adult intussusception is rare, accounting for 1 in 1300 abdominal operations, while intussusception in childhood is much more common, especially in the first year of life [10-11]. Most cases of pediatric intussusception, 75% to 90%, are idiopathic, and no causative agent is discovered [10-11]. However, recent investigations have supported viral infections, most commonly adenovirus C, as a source of intussusception in children [3,10]. Intussusception occurs more frequently in the small bowel, and the ileocolic junction has the highest reported incidence [10,12]. Lead points are anatomic or structural pathologic variables serving as the origin and impetus for intussusception [11]. A viral etiology is less common in adults; however, lead points are more frequently discovered in adults and are often neoplasms [11].

Episodic abdominal pain is the primary symptom of intussusception in adults and children [10-11]. Nausea and bilious vomiting are often present [10-12]. In a minority of cases when ischemia occurs, hematochezia may be present and is often characterized as "red currant jelly" stool due to the mixture of blood and mucus from the sloughing of the ischemic mucosa [10]. In severe, advanced presentations, intestinal perforation, necrosis, and peritonitis may occur, resulting in fever and symptoms of sepsis [10-11]. Diffuse or localized abdominal pain on palpation is often present on physical examination. A mass is sometimes palpated on the exam, mostly in the right upper abdominal quadrant, and is frequently described as "sausage-shaped" [10]. A "dance sign" has been associated with intussusception and is an empty or scaphoid appearance in the right lower abdominal quadrant relative to and just inferior to the sausage-shaped mass [10]. These signs may be present in children but infrequently appear in adults [11].

Imaging studies are required to diagnose intussusception definitively. In children, abdominal ultrasonography is the preferred modality and classically reveals the doughnut or target sign produced by transverse visualization of the telescoping bowel [10]. In adults, contrast-enhanced CT is recommended to assess for intussusception [11]. CT scans are more sensitive than ultrasounds for adult intussusception and have the benefit of ascertaining pathologic lead point neoplasms, to which adult intussusception is more often attributed [11]. CT scans can visualize signs of ischemia, predict favorable outcomes of intussusception, and are superior at recognizing possible alternate diagnoses in adults who possess a broader differential for acute abdominal pain than children [11].

Management principles vary between cases of intussusception in children and adults. In pediatric populations, air or liquid contrast enemas are both diagnostic and therapeutic for most patients [10-14]. Both air and liquid enemas are effective; however, recent investigations have found improved success rates with pneumatic enemas and similar rates of perforation complications [15]. Supportive care with fluid administration, antiemetics, and analgesia is recommended, and there is mixed evidence for adjunctive steroid administration [11-12]. Antibiotics should be reserved for suspected sepsis [11-12]. Enema decompression therapy has a high resolution rate of around 80%; however, treatment failure may indicate the need for surgical management [10-12]. In adults, treatment of intussusception is mostly surgical due to the high rates of malignant lesions that serve as etiologic lead points for intussusception [11-12].

Complications from intussusception include bowel obstruction, ischemia, necrosis, perforation, peritonitis, and sepsis, which are all much more common in adults than children, possibly due to the difficulty of early diagnosis in adults [10-11]. Tumor seeding is a possible adverse event related to surgical intervention in adults with intussusception secondary to malignancy [11]. The prognosis is favorable in children but poorer in adults, likely attributable to the challenges of early diagnosis and the high rates of malignancy [10-12].

The presented case is challenging due to its rarity and the complex intersection between two disorders, varicella infection and intussusception, which are generally considered distinct and unrelated. A well-known relationship between previous iterations of the rotavirus vaccination and intussusception has long been observed and may have given an earlier clue to the association between viruses and intussusception [16-17]. The extracutaneous expression of varicella infection, including gastrointestinal complications, has long been observed. In our case, we observed transaminitis, leukopenia, thrombocytopenia, and pneumonia, all in addition to intussusception and resolving with antiviral treatment. The patient's elevated liver enzymes could be attributed to acute VZV infection; however, they may also represent chronic metabolic dysfunction-associated liver disease as the patient is obese and diabetic, and imaging demonstrates fatty infiltration. In this case, leukopenia may be associated with a response to varicella zoster or may indicate an underlying immunosuppression that made the patient more susceptible to adult varicella zoster infection. The thrombocytopenia was transient and quickly resolved following treatment, suggesting it was likely an acute response to varicella zoster. One study spanning five years in a New York City hospital in 1935 investigated 2,534 patients hospitalized with varicella and observed multisystem manifestations, including bacterial skin infections, pneumonia, encephalitis, and hematologic problems, as well as gastrointestinal complications in eight patients [18]. While the exact mechanism of virally induced intussusception is unknown, several logical conclusions can be drawn from our knowledge of the two disorders. Enlarged mesenteric lymph nodes secondary to viral infections may serve as lead points for the invagination of the intestine into the proximate bowel [19]. Virally induced hypertrophy of the small intestine's lymphoid Peyer's patches may precipitate intussusception [19]. VZV specifically has demonstrated ganglionitis and neuropathic enteric nervous system dysfunction, which may cause intussusception through discoordinated peristalsis [19-21]. In one case study of varicella-induced intussusception, histopathologic investigation of intestinal tissue confirmed the presence of VZV ribonucleic acid in myenteric ganglia and submucosal nerve fiber-like tissue [19]. New evidence continues implicating a viral cause for intussusception and may support an even more significant attribution than previously thought. An ecological study in Australia found that the decrease in communicable diseases secondary to COVID-19 lockdowns resulted in a significant decrease in intussusception cases, much more so than would be expected based on our current understanding of infection-induced intussusception [22]. Some case studies implicate the COVID-19 virus as a cause of intussusception [23]. One analysis of intussusception cases in children under two years of age in four Asian countries revealed a statistically significant correlation between adenovirus C infection and intussusception and that the higher viral loads were associated with increased risk [3]. This study also found that human herpes virus 6, another member of the Herpesviridae family like varicella and the causative agent of roseola, was linked to intussusception [3]. At this point, case studies describing varicella-induced intussusception comprise most of our knowledge about the phenomenon, and further investigation is needed.

## Conclusions

Understanding the diverse manifestations of VZV, including rare gastrointestinal presentations like intussusception, is critical. Early recognition and antiviral therapy are key to managing complications and preventing systemic dissemination. This case highlights the importance of maintaining a broad differential diagnosis in adult patients presenting with abdominal pain and concurrent rash. Further research is needed to elucidate the connection between viruses like VZV and intussusception.
